# Systematic study of genes influencing cellular chain length in *Streptococcus sanguinis*

**DOI:** 10.1099/mic.0.071688-0

**Published:** 2014-02

**Authors:** Karra Evans, Victoria Stone, Lei Chen, Xiuchun Ge, Ping Xu

**Affiliations:** 1 VCU Philips Institute of Oral and Craniofacial Molecular Biology, Virginia Commonwealth University, Richmond, VA 23298-0566, USA; 2 Center for the Study of Biological Complexity of Virginia Commonwealth University, Richmond, VA, USA; 3 Department of Microbiology and Immunology, Virginia Commonwealth University, Richmond, VA, USA

## Abstract

*Streptococcus sanguinis* is a Gram-positive bacterium that is indigenous to the oral cavity. *S. sanguinis*, a primary colonizer of the oral cavity, serves as a tether for the attachment of other oral pathogens. The colonization of microbes on the tooth surface forms dental plaque, which can lead to the onset of periodontal disease. We examined a comprehensive mutant library to identify genes related to cellular chain length and morphology using phase-contrast microscopy. A number of hypothetical genes related to the cellular chain length were identified in this study. Genes related to the cellular chain length were analysed along with clusters of orthologous groups (COG) for gene functions. It was discovered that the highest proportion of COG functions related to cellular chain length was ‘cell division and chromosome separation’. However, different COG functions were also found to be related with altered cellular chain length. This suggested that different genes related with multiple mechanisms contribute to the cellular chain length in *S. sanguinis* SK36.

## Introduction

*Streptococcus sanguinis*, previously known as *Streptococcus sanguis*, is a Gram-positive facultative anaerobe belonging to the viridans group of streptococci (Kilian & Holmgren, 1981; Kilian *et al.*, 1989; Xu *et al.*, 2007; Truper & De’clari, 1997). The cells of *S. sanguinis* grow in chains and clusters that directly bind to the salivary pellicle of the tooth surface. Using different mechanisms of attachment, *S. sanguinis* serves as a tether for the colonization of other micro-organisms. Oral micro-organisms then organize and cooperate to develop biofilms on dental surfaces. The formation of this biofilm plays an essential role in the establishment of periodontal disease (Kolenbrander, 2000; Kolenbrander & London, 1993; Socransky *et al.*, 1977; Xu *et al.*, 2007; Truper & De’clari, 1997). *S. sanguinis* is also one of the recognized causative agents of infective endocarditis when it enters the bloodstream, which can lead to serious health complications (Ahmed *et al.*, 2003).

One factor that may contribute to *S. sanguinis* biofilm formation and endocarditis virulence is cellular chain length. For instance, the length of the chain can affect the physical behaviour of cells in biofilm formation. The longer the cell chains are, the easier it is for cells to aggregate with each other. Cellular chain length was shown to be related to bacterial invasion in *Streptococcus pneumoniae* (Dalia & Weiser, 2011) and was also found to have a negative correlation with phagocytosis susceptibility in *Streptococcus mutans* (Nakano *et al.*, 2005; Wen & Burne, 2002). Cellular chain formation is dependent on the geometry of cell division during binary fission. Cellular division occurring within two planes perpendicular to each other results in a tetrad formation of daughter cells, while divisions that occur within three planes result in the arrangement of daughter cells in an eight member cubic structure (Zapun *et al.*, 2008). Irregular patterns of cell division that occur in random planes result in clustered bunches of bacteria, while a regular pattern of cell division occurring along one plane or axis results in the formation of cellular chains (Zapun *et al.*, 2008). In streptococci, cell division usually occurs along one plane and results in chains composed of two or more cells. However, as streptococcal cellular chain length is influenced by a number of factors, it should be noted that cellular morphology and chain formation in streptococci are highly variable and are known to differ from strain to strain (Higgins *et al.*, 1970; Nakano *et al.*, 2005; Thibodeau & Ford, 1991).

Chain formation in *S. sanguinis* has been shown to affect competitiveness altering endocarditis virulence (Turner *et al.*, 2009). Some studies predict conditions that may affect cellular chain length may also affect adhesion (Murchison *et al.*, 1982) and aggregation (Murchison *et al.*, 1982; Nakano *et al.*, 2005), which can contribute to virulence.

The streptococcal cellular chain length and morphology are influenced by a number of factors. Previous studies suggest that cell wall components such as peptidoglycan, lipoteichoic acids and cell wall anchor proteins greatly affect the morphology of cells (Thibodeau & Ford, 1991). Factors such as environmental conditions or genetic and chemical mutations that control or affect these cellular components can contribute to streptococcal cell adhesion and morphology (Higgins *et al.*, 1970; Nakano *et al.*, 2005; Thibodeau & Ford, 1991). We recently created a comprehensive bank of gene deletion mutants in *S. sanguinis* SK36 strain (Xu *et al.*, 2011). In the present study, using phase-contrast microscopy, we analysed morphological changes of these gene deletion mutants and identified a number of genes related to cellular chain length.

## Methods

### 

#### Bacterial strains and mutants.

*S. sanguinis* SK36 single gene deletion mutants with a promoterless kanamycin cassette and complemented strains with a promoterless erythromycin cassette were previously constructed using a PCR-based recombinant method as described (Xu *et al.*, 2011). Mutants are indicated by ‘Ssx’ followed by their corresponding gene ‘SSA’ numbers.

*S. sanguinis* gene deletion mutants were cultured in brain heart infusion (BHI) broth (BD Sciences) supplemented with kanamycin (Fisher Scientific) to 500 µg ml^−1^ using 1 ml 96-deep-well plates (Fisher Scientific). Deletion mutants from frozen stock plates were transferred to 96-deep-well plates. The mutants were grown overnight at 37 °C under microaerobic conditions (6 % O_2_, 7.2 % CO_2_, 7.2 % H_2_ and 79.6 % N_2_) (Xu *et al.*, 2007; Chen *et al.*, 2011). Slower growing mutants were cultured for 2 days to allow for complete growth. The failed growing mutants were re-inoculated no more than twice. Each 96-deep-well plate contained approximately 90 mutants, two wells contained wild-type strain SK36 (as controls) and four wells contained sterile BHI medium to monitor cross contamination. Except for BHI supplemented with 10 µg ml^−1^ erythromycin, the conditions for culturing complemented strains were the same as above.

#### Phase-contrast microscopy.

Cellular morphology of each deletion mutant and wild-type of *S. sanguinis* SK36 was observed using phase-contrast microscopy. Samples were carefully collected from each well of a 96-deep-well plate, avoiding disruption of the cellular chains and clusters. Clustered mutants or cultures that displayed excessive growth were diluted twofold with BHI prior to observation.

Each mutant was examined at ×200 total magnification using a Carl Zeiss AxioVision microscope. To determine cellular chain length, 50 chains were randomly selected and measured using the AxioVision Le Rei 4.3 program. The geometric mean of the 50 measured chains for each mutant was compared with the geometric mean of the SK36 control. Student’s *t*-test was used to determine significant differences among the cellular chain lengths. The Bonferroni correction was used to adjust for the comparison of mutants in the 96-well plates and the total number of mutants being evaluated. Therefore, mutants that were shorter or longer than SK36 (13.86–17.36 µm) with a *P*-value <0.0005 were considered to be short or long.

#### Auto-aggregation assay.

The auto-aggregation of *S. sanguinis* wild-type, mutants and their complemented strains was determined as described by Luo *et al.* (2008). Briefly, each overnight culture in BHI medium was mixed, transferred into a 1 ml cuvette and kept at room temperature. The absorbance of the culture at 600 nm was measured at 0 h (*A*_0_) and 8 h (*A*_8_) using a BioMate spectrophotometer (Thermo Scientific). Auto-aggregation ability was expressed as auto-aggregation percentage (Ag%) and calculated using Ag% = [(*A*_0_−*A*_8_)/*A*_0_]×100.

#### Gene function assignment.

To determine putative gene function, protein sequences were searched against the previously annotated *S. sanguinis* SK36 genome (Xu *et al.*, 2007). The cluster of orthologous groups (COG) annotation was downloaded from the NCBI database and pathways were analysed via the Kyoto Encyclopedia of Genes and Genomes (KEGG), combined with manual curation and validation. Once each mutant was examined and measured, its putative gene function and COG were recorded. The ratio of the number of cellular chain length mutants for each COG and the total number of non-essential gene mutants belonging to the COG was calculated.

#### Scanning electron microscopy (SEM).

Following overnight incubation, 0.5 ml of each culture was deposited onto a 0.1 µm disposable Millipore filter to remove BHI broth. The samples were fixed using 2 % glutaraldehyde in 0.1 M sodium cacodylate buffer, followed by 1 % osmium tetroxide in 0.1 M sodium cacodylate buffer. Samples embedded in the filters were then dehydrated in ethanol and hexamethyldisilazane (HMDS) and finally allowed to air-dry. The filters were sectioned and mounted. Prior to visualization samples were coated with gold for 3 min (EMS-550 Automated Sputter Coater; Electron Microscopy Sciences). Micrographs were taken at ×20 000, ×30 000 and ×40 000 total magnification using a ZEISS EVO50 XVP scanning electron microscope (Carl Zeiss).

#### Microarray analysis.

RNA samples were isolated from exponential phase cells (OD_660_ 0.6–1.0) of the two-component system (TCS) gene mutants. RNA samples from SK36 strain cells were used as the control. RNA isolations were performed in triplicate. Microarray analysis was performed as previously described (Xu *et al.*, 2011). Briefly, RNA was isolated with the RNeasy mini kit (Qiagen) from cells lysed by lysozyme treatment and mechanical disruption. DNA was removed from the RNeasy mini kit column by DNase I treatment. RNA samples were labelled by Cy3 or Cy5 in cDNA synthesis. The labelled cDNA of the test and the control samples was mixed and hybridized with the *S. sanguinis* SK36-spotted microarray version 2 (produced by the Pathogen Functional Genomics Resource Center at J Craig Venter Institute). Following hybridization and scanning, signals were calculated, normalized and analysed statistically to identify modulations in transcriptional activity. The microarray data were deposited in the NCBI Gene Expression Omnibus (GEO) with access number GSE48940.

## Results

### Collection of longer and shorter chain mutants

**Fig. 1.**
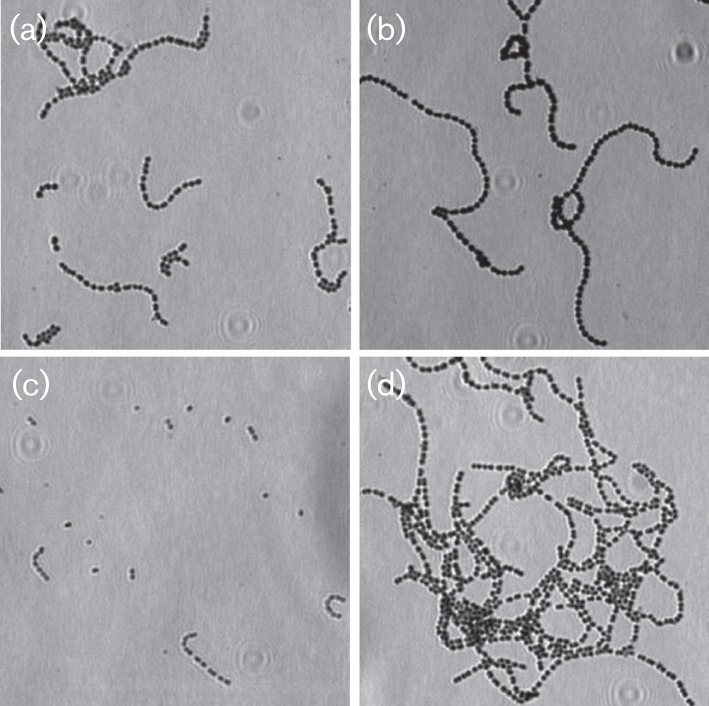
Chain morphologies of mutants. (a) SK36, the wild-type strain. (b) A longer chain mutant with deletion of SSA_0933 gene for acetyltransferase. (c) A shorter chain mutant with deletion of SSA_0422 gene for phosphoglycerate mutase family protein. (d) A chain clustered mutant with deletion of SSA_0285 gene for formate acetyltransferase 3.

We first classified four basic mutant groups based on the chain morphology observed: longer, shorter, clustered and normal chains. Fig. 1 shows the different morphologies of three mutants compared with the wild-type *S. sanguinis* SK36 strain.

**Fig. 2.**
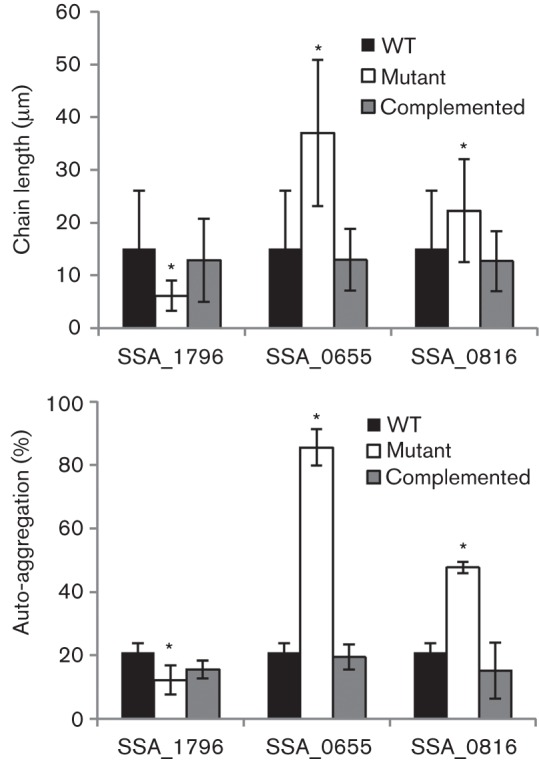
Cellular chain length and auto-aggregation of selected mutants and their complementation. Upper panel, the cellular chain lengths of mutants and their complemented strains were examined and compared with the wild-type (WT) as described in Methods. Lower panel, their auto-aggregations were determined by OD_600_ and the aggregation percentage was calculated as described in Methods. * *P*<0.05. Error bars indicate ±sd.

Each mutant was observed using phase-contrast microscopy. Three to four images were photographed for length analysis. Initially, images of the mutants were visually inspected to select those with different cellular morphology (longer, shorter or clustered chains) from the wild-type strain, SK36, in the same 96-well culture plate. The cellular chain lengths of the selected mutants were then carefully measured. Fifty chains were randomly selected from the images and manually measured using the AxioVision Le Rei 4.3 program. The statistical significance of mutant cellular chain lengths compared with SK36 was calculated. Over 300 of 2000 mutants, or approximately 15 % of genes in the genome, were identified to have significant changes in the morphological cellular chain length. Of these, 156 mutants had longer chains and 151 mutants had shorter chains (Table S1, available in the online Supplementary Material). In Table 1, we list the 20 longest cellular chain mutants and in Table 2, the 20 shortest cellular chain mutants. From the top 20, we picked the three mutants Ssx_1796, Ssx_0655 and Ssx_0816 with shorter, longer and clustered phenotypes, respectively, to create their complemented strains. The cellular chain length of these complemented strains was recovered to the same level as the wild-type (Fig. 2). By examining these longer and shorter mutants, we found 27 mutants showed clustered cellular morphology. These clusters were not dissolved by twofold BHI dilution. Comparing these clustered mutants with cellular chain length, we found that all of the clustered mutants formed longer chains. In addition, we examined the auto-aggregation of the mutants Ssx_1796, Ssx_0655 and Ssx_0816. The results showed that auto-aggregation ability was significantly reduced in Ssx_1796 with shorter chain length and increased in Ssx_0655 with longer chain length as well as in Ssx_0816 with clustered chains compared with the wild-type (Fig. 2). However, the auto-aggregation ability was recovered in their complemented strains. These results suggest a correlation between cellular chain length and bacterial cell aggregation.

**Table 1.** Twenty longest cellular chain length mutants See Table S1 for complete mutant list.

Mutant	*L* (µm)*	*P*-value†	Product	COG	KEGG
Ssx_0655	37.02	2.23e−16	Cell division protein FtsA	Cell division and chromosome partitioning	
Ssx_0499	32.65	1.44e−15	ABC-type dipeptide transport system, periplasmic component	Amino acid transport and metabolism	ABC transporter
Ssx_1285	27.42	1.39e−08	Hypothetical protein		
Ssx_1270	27.39	7.11e−08	Flavodoxin	Energy production and conversion	
Ssx_1306	27.09	3.99e−06	Trk transporter NAD^+^ binding protein – K^+^ transport	Inorganic ion transport and metabolism	
Ssx_1958	26.82	8.84e−07	Adaptor protein		
Ssx_1266	26.66	5.64e−07	CrcB-like protein 1	Cell division and chromosome partitioning	
Ssx_0933	26.44	4.60e−07	Acetyltransferase	General function prediction only	
Ssx_0860	25.80	8.71e−06	*N*-Acetylmuramidase/lysin	Cell envelope biogenesis, outer membrane	
Ssx_0908	25.69	3.83e−06	ABC-type uncharacterized transport system, periplasmic component	General function prediction only	
Ssx_0504	25.68	1.33e−06	Peptide ABC transporter, ATP-binding protein	Amino acid transport and metabolism/inorganic ion transport and metabolism	ABC transporter
Ssx_0881	25.63	2.72e−05	Putative lipoprotein		
Ssx_0963	25.53	1.60e−09	Peptidoglycan *N*-acetylglucosamine deacetylase A		
Ssx_2224	25.43	1.61e−11	Phosphotyrosine-protein phosphatase	Carbohydrate transport and metabolism/cell envelope biogenesis, outer membrane	
Ssx_1277	25.32	9.49e−07	d-Alanyl-d-alanine carboxypeptidase		
Ssx_0816	25.20	6.44e−07	Copper transport operon or penicillinase transcription repressor	Transcription	Transcriptional regulator
Ssx_2146	25.07	1.92e−08	Metallo-β-lactamase	General function prediction only	
Ssx_0144	25.01	5.14e−05	TetR family transcriptional regulator	Transcription	Transcriptional regulator
Ssx_0207	24.97	6.31e−07	Hypothetical protein		
Ssx_1268	24.96	2.66e−06	Hypothetical protein	Amino acid transport and metabolism	Phenylalanine, tyrosine and tryptophan biosynthesis

* *L*, cellular chain length.

† *P*-value for statistically significant difference compared with SK36.

**Table 2.** Twenty shortest cellular chain length mutants See Table S1 for complete mutant list.

Mutant	*L* (µm)*	*P*-value†	Product	COG	KEGG
Ssx_1116	5.01	3.52e−24	Hypothetical protein		
Ssx_1796	5.32	1.69e−24	Transcription elongation factor GreA	Transcription	Transcriptional regulator
Ssx_1103	5.86	5.08e−20	Hypothetical protein		
Ssx_1110	6.10	5.97e−18	Hypothetical protein		
Ssx_2312	6.12	3.02e−22	Hypothetical protein		
Ssx_2147	6.20	1.32e−18	Hypothetical protein		
Ssx_0105	6.25	6.54e−14	Uridine kinase	Nucleotide transport and metabolism	Pyrimidine metabolism
Ssx_2296	6.38	1.88e−21	XRE family transcriptional regulator	Transcription	Transcriptional regulator
Ssx_2077	6.40	2.13e−15	Hypothetical protein	General function prediction only	
Ssx_2337	6.41	7.03e−19	Hypothetical protein	Function unknown	
Ssx_2152	6.43	1.10e−15	ABC-type transporter (uncharacterized), ATPase component	General function prediction only	ABC transporter
Ssx_2333	6.51	9.01e−17	Integral membrane protein	Cell envelope biogenesis, outer membrane	
Ssx_2250	6.52	8.40e−18	Peptide ABC transporter permease	Function unknown	
Ssx_0773	6.64	9.18e−15	PTS enzyme I	Carbohydrate transport and metabolism	Phosphotransferase system (PTS)
Ssx_2332	6.65	1.54e−18	d-Alanine-poly(phosphoribitol) ligase subunit 2	Lipid metabolism/secondary metabolites biosynthesis, transport and catabolism	d-Alanine metabolism
Ssx_1860	6.69	3.60e−16	Penicillin-binding protein 1A	Cell envelope biogenesis, outer membrane	Peptidoglycan biosynthesis
Ssx_2285	6.77	1.40e−15	Hypothetical protein		
Ssx_2162	6.79	1.17e−12	Hypothetical protein		
Ssx_0148	6.83	7.10e−14	Sugar ABC transporter, ATP-binding protein	Carbohydrate transport and metabolism	ABC transporter
Ssx_2141	6.83	3.19e−14	Argininosuccinate lyase	Amino acid transport and metabolism	Alanine, aspartate and glutamate metabolism

* *L*, Cellular chain length.

† *P*-value for statistically significant difference compared with SK36.

### Longer chains related with cell division and chromosome partition

**Fig. 3.**
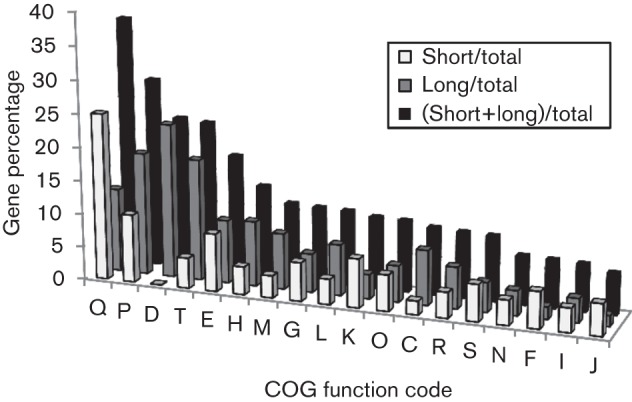
Cellular chain length related gene distribution by COG functions. The ratio between the number of longer chain or shorter chain mutants and the total number of annotated genes in the genome (gene percentage) was calculated for every COG function. COG function abbreviations: Q, secondary metabolites biosynthesis, transport and catabolism; P, inorganic ion transport and metabolism; D, cell division and chromosome partitioning; T, signal transduction mechanisms; E, amino acid transport and metabolism; H, coenzyme metabolism; M, cell envelope biogenesis, outer membrane; G, carbohydrate transport and metabolism; L, DNA replication, recombination and repair; K, transcription; O, post-translational modification, protein turnover, chaperones; C, energy production and conversion; R, general function prediction only; S, function unknown; N, cell motility and secretion; F, nucleotide transport and metabolism; I, lipid metabolism; J, translation, ribosomal structure and biogenesis.

To analyse the longer and shorter chained mutants, we collected annotated gene functions for each mutant. Of the 307 cellular chain length related genes, 108 genes were annotated as hypothetical proteins. This suggested that many of these gene functions were new. To study functional categories, we examined gene assignments in COGs. COGs are delineated by comparing protein sequences encoded in complete genomes, representing major phylogenetic lineages in at least three different genomes (Tatusov *et al.*, 2001). COG defines 18 different categories, including ‘information storage and processing’, ‘cellular processes’ and ‘metabolism’ functional annotations. Of 307 cellular chain length related mutants, 199 genes have annotated functions, including 127 with COG assignments. To profile the 127 cellular chain length related mutants by COG assignments, we compared the proportions of their assigned gene functions in COG. As indicated in Fig. 3, multiple COG functions were shown to relate with cellular chain length. This suggested that cellular chain length might present a complex microbial phenotype, relying on a number of genes and their interactions to yield the observed phenotypes. We then compared the proportions of COG functions for longer and shorter chains. This showed that analysis of both longer and shorter chains together resulted in the highest ratio of COG functions as ‘secondary metabolites biosynthesis, transport and catabolism’, followed by ‘inorganic ion transport and metabolism’. However, analysis of longer and shorter chains separately resulted in the highest ratio for longer chain mutants as ‘cell division and chromosome partitioning’, while the highest ratio for shorter chain mutants was ‘secondary metabolites biosynthesis, transport and catabolism’. Mutants with functions of ‘cell division and chromosome partitioning’ were all longer-chained.

### TCSs related to cellular chain length

Interestingly, 12 genes related to cellular chain length were involved in ‘signal transduction mechanisms’ of COG annotation. Therefore, we examined mutants deficient in TCSs for their cellular morphology. TCSs are commonly found in bacteria and are composed of a membrane bound protein called a histidine kinase (HK) that senses environmental changes and autophosphorylates its own histidine residue. The HK signals to its corresponding cytosolic response regulator (RR) protein by transferring the phosphate to an aspartate residue. The RR undergoes a conformational change and induces a response by regulating gene transcription. Of all 29 genes for 15 TCSs in the *S. sanguinis* genome, we found that seven of the gene mutants showed statistical differences in cellular chain length morphology, including three HK – SSA_0205, SSA_0517 and SSA_1120 – and four RR – SSA_0217, SSA_1113, SSA_1119 and SSSA_1972. All seven TCS gene mutants formed longer chains (Table S1).

To determine if cellular chain length could be attributed to transcriptional regulation controlled by TCS response regulators and to find the cellular chain length related regulons, we performed microarray analysis to examine their gene expression profile. We selected mutant Ssx_1972 for further microarray analysis, which exhibited the longest cellular chain length phenotype in the four TCS RR mutants (Table 3). For 2270 total gene expressions in Ssx_1972, there were 119 genes downregulated and 111 genes upregulated by a twofold cut-off compared with SK36 (Table 3). We analysed the possible association of up- and downregulated genes for their contributions to the longer chain phenotype of Ssx_1972. Because the deletion of gene SSA_1972 in mutant Ssx_1972 showed a longer chain phenotype, we defined a gene whose mutant showed a longer chain and a decreased gene expression in Ssx_1972 as a positively related gene with SSA_1972. There were seven positively related genes, exhibiting longer chains in their mutants with downregulated gene expressions in Ssx_1972. These genes were SSA_0209, SSA_0531, SSA_0588, SSA_0724, SSA_0737, SSA_1275 and SSA_1284 (Table 3). In contrast, there were eight negatively related genes displaying shorter chains in their mutants with upregulated gene expressions in Ssx_1972. These genes were SSA_0759, SSA_0760, SSA_1715, SSA_1897, SSA_2117, SSA_2141, SSA_2159 and SSA_2337. All positively and negatively related genes should contribute to the cellular chain length of Ssx_1972. However, there were also downregulated genes showing shorter chain mutants (SSA_0142, SSA_0222, SSA_0393, SSA_1907, SSA_2077, SSA_2250) and upregulated genes showing longer chain mutants (SSA_0191, SSA_0215, SSA_0217, SSA_0678, SSA_0758, SSA_1218, SSA_1266, SSA_1900, SSA_2224, SSA_2233). This complicated result suggests that the longer chain of Ssx_1972 might be a comprehensive result of multiple gene regulations. This information again suggests multiple genes may be involved in cellular chain length formation.

**Table 3.** Gene expressions of shorter or longer chain genes in Ssx_1972, a longer chain mutant, compared with SK36

Locus	Gene	Product	Expression*	sd	Short†	Long†
SSA_0222	–	PTS system, mannose-specific IID component	0.1210	0.0583	Y	
SSA_0393	–	Bacteriocin ABC transporter permease/ATP-binding protein	0.3499		Y	
SSA_1907	–	Hypothetical protein SSA_1907	0.3932	0.0167	Y	
SSA_2250	–	Peptide ABC transporter permease	0.4245	0.0125	Y	
SSA_0142	–	Hypothetical protein SSA_0142	0.4440	0.1035	Y	
SSA_2077	–	Hypothetical protein SSA_2077	0.4932	0.0512	Y	
SSA_0759	*argB*	Acetylglutamate kinase	3.1950	0.6376	Y	
SSA_0760	–	Acetylornithine aminotransferase	9.4869	4.9372	Y	
SSA_1715	*serC*	Phosphoserine aminotransferase	2.0630	1.0319	Y	
SSA_1897	–	Hypothetical protein SSA_1897	2.2604	0.6261	Y	
SSA_2117	*rmuC*	DNA recombination protein RmuC	2.5179	0.7197	Y	
SSA_2141	*argH*	Argininosuccinate lyase	4.6751	3.6790	Y	
SSA_2159	–	Hypothetical protein SSA_2159	4.6105	1.8498	Y	
SSA_2337	–	Hypothetical protein SSA_2337	2.0824	0.5594	Y	
SSA_0724	–	Multidrug ABC transporter ATPase/permease	0.3345			Y
SSA_0209	*pepA*	Glutamyl aminopeptidase	0.4287	0.0893		Y
SSA_0737	*sagP*	Arginine deiminase	0.4317	0.1570		Y
SSA_0588	–	l-Cystine ABC transporter, substrate-binding component	0.4475	0.1851		Y
SSA_1284	–	Hypothetical protein SSA_1284	0.4670			Y
SSA_1275	–	Hypothetical protein SSA_1275	0.4830	0.2878		Y
SSA_0531	*eutQ*	Ethanolamine utilization protein EutQ	0.4985	0.4339		Y

* Relative expression in Ssx_1972 compared with SK36 using microarray analysis.

† Y, Yes.

### Ultrastructure of cellular chain length related mutants by SEM

**Fig. 4.**
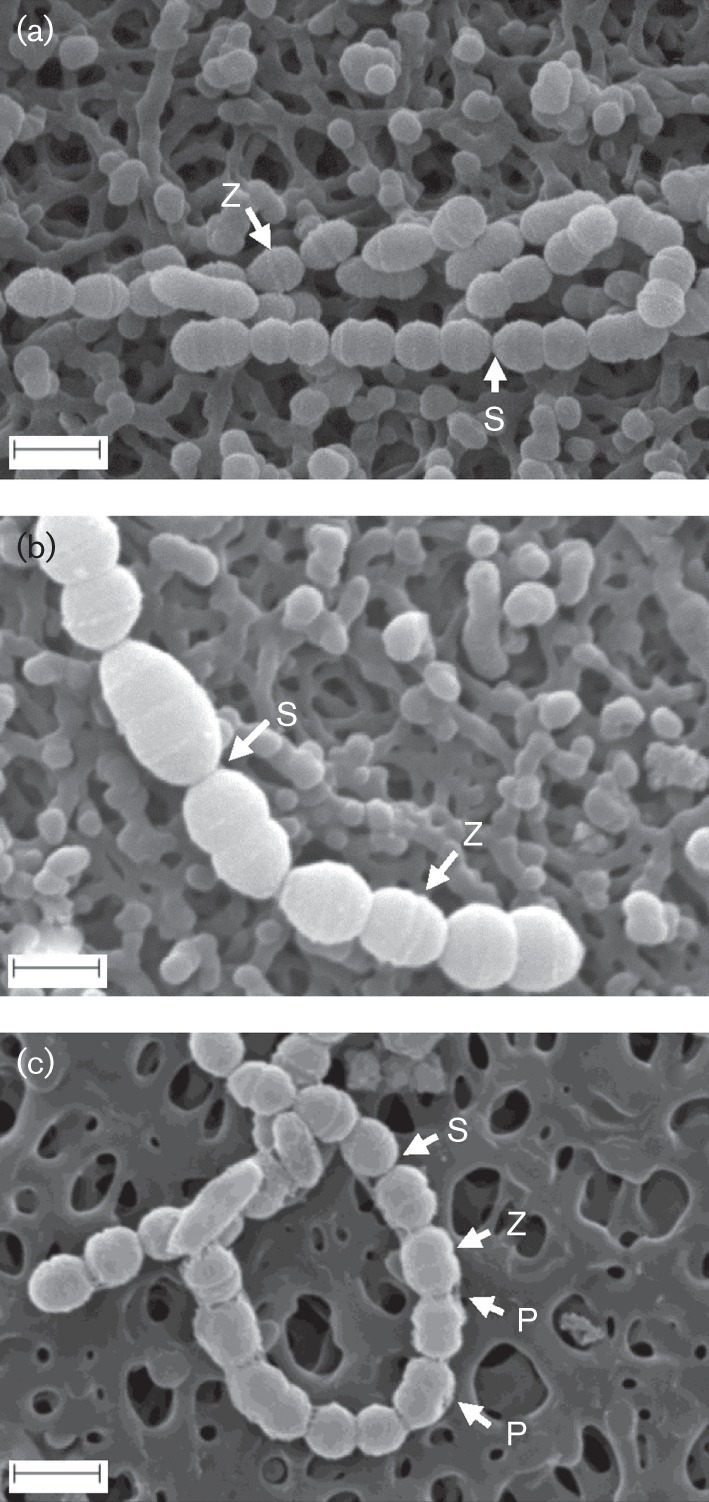
Cell morphological comparisons by SEM. (a) The wild-type strain SK36. (b) Ssx_0655, a cell division protein FtsA mutant, shows wider and longer cells. (c) Ssx_2223, a capsular polysaccharide biosynthesis protein Wzd (chain length regulator) mutant, shows clear capsule surrounding the cells. Septum (S), Z-ring (Z) and polysaccharide (P) are indicated. Bars, 1 μm.

To further examine longer chain mutants, we used SEM to observe the fine cellular surface morphology and ultrastructure. We selected two mutants, Ssx_0655 and Ssx_2223, for further examination. Ssx_0655 formed the longest chain compared with the other cellular chain length mutants (Tables 1 and S1) and SSA_2223 is a homologue of a reported chain length regulator, Wzd. Interestingly, SSA_0655 encoded the FtsA protein, which is an early component of the Z-ring, the structure that divides most bacteria, formed by tubulin-like FtsZ (Szwedziak *et al.* 2012), and plays an important role in cell division. SSA_2223 is annotated to be involved in capsular polysaccharide biosynthesis. The ultrastructure of Ssx_0665 and Ssx_2223 displayed larger cells and a thicker capsule to keep more cells in the chain. Ssx_0655 appeared to have wider and longer cells than SK36 (Fig. 4). Although Ssx_0655 cells still formed ring structures (Z-rings) for division, the cell shape was longer and fatter than SK36. For the Ssx_2223 mutant, there was a clear sheath surrounding the cells, which indicated more capsule present (Fig. 4). The thick sheath maintained cells in longer chains.

## Discussion

A systems biology approach was made possible by the availability of the genome-wide mutants of the *S. sanguinis* SK36 strain. In this study, we examined nearly all non-essential gene mutants previously created (Xu *et al.*, 2011) for their possible involvement in cellular chain length. We identified over 300 mutants which showed statistically significant differences in length from SK36. It should be noted that our cellular chain length list was not complete. Some mutants may have been missed in this large-scale analysis for different reasons. For example, some mutants failed to grow after two inoculations from stocks and some were not recognized as having an altered cellular chain length through eyeball visualization and were missed in the final analysis. However, the analysis of identified mutants still presents meaningful insight into the association of gene functions and cellular chain length due to the large number of mutants analysed. Based on COG analysis, the highest gene ratio for longer chain mutants was ‘cell division and chromosome partitioning’ (Fig. 3). Three of the longer-chained mutants had deleted genes (SSA_0655, SSA_1266, SSA_2222) related to this function (Tables 1 and S1, COG code D).This finding was not surprising as the ability of cells to divide and separate would be an inherent factor contributing to cellular chain length. Chain formation in streptococci is the result of proper cell division that occurs on one plane in a designated area of the cell (Lara *et al.*, 2005; Zapun *et al.*, 2008). One of those longer chain mutants was Ssx_0655, whose deleted gene was annotated as cell division protein FtsA. The *ftsA* gene belongs to a cluster of cell division and cell wall synthesis genes in many bacteria and is important for septation although its exact role is unclear. In *Escherichia coli*, overexpression or deletion of the *ftsA* gene inhibits normal cell septation, which is attributed to the formation of longer septate filaments (Wang & Gayda, 1990). In *Bacillus subtilis, ftsA* mutations cause an abnormal Z-ring formation due to the loss of the recruitment of cell division proteins necessary for correct Z-ring assembly (Jensen *et al.*, 2005). The FtsA protein localizes to the equatorial zones of dividing cells where it interacts with FtsZ, a tubulin-like protein, forming the septal ring (Lara *et al.*, 2005). In *S. sanguinis*, the *ftsZ* gene was found to be essential so we were not able to examine its mutant morphology (Xu *et al.*, 2011). Although the *ftsA* gene is not essential; its absence has been observed to have a significant effect on cell division and chain morphology. We hypothesized that longer chains are formed by enlarged cells that do not properly divide. SEM analysis (Fig. 4a) showed that individual cells of the Ssx_0655 mutant were larger than wild-type SK36 due to a defect in cell septation. The two other longer chain mutants whose genes were related with ‘cell division and chromosome partitioning’ in COG were SSA_1266 and SSA_2222. SSA_1266 encodes a camphor resistance protein, CrcB. CrcB, an integral membrane protein, may be involved in chromosome condensation (Sand *et al.*, 2003). Several genes in the same operon, including SSA_1267, SSA_1268, SSA_1269 and SSA_1270, all showed the same longer chain morphology, suggesting they share a similar function with SSA_1266. SSA_2222 has been annotated as Wze, a cytoplasmic tyrosine kinase. SSA_2222 may be responsible for the phosphorylation of its neighbouring gene product, SSA_2223. SSA_2223, annotated as Wzd, is a chain length regulator involved in capsular polysaccharide biosynthesis. SSA_2222 may control polysaccharide biosynthesis through phosphorylation of tyrosine residues on its neighbouring gene product SSA_2223. In turn, phosphorylation of tyrosine residues in SSA_2223 changes the enzyme activity in capsular polysaccharide biosynthesis. The mutant of SSA_2223 also showed longer chain morphology (Table S1, Fig. 4). It has been reported that Wze interacts with Wzd to regulate the rate of capsule polysaccharide biosynthesis in *Streptococcus thermophilus* (Cefalo *et al.*, 2011). It has also been reported that Wzd- and Wze-encoding genes are co-expressed and their interactions are required for synthesis of capsular polysaccharide at the division septa in *S. pneumoniae* (Henriques *et al.*, 2011). The mutant of SSA_2224 which showed a longer chain phenotype may also control SSA_2223, similar to SSA_2222. SSA_2224 encodes a phosphotyrosine-protein phosphatase. Although the three genes (SSA_0655, SSA_1266 and SSA_2222) are involved in different mechanisms they are all in COG classification ‘cell division and chromosome partitioning’. SSA_0655 is involved in cell division and cell wall synthesis, SSA_1266 is involved in chromosome condensation and SSA_2222 is involved in the synthesis of capsular polysaccharide. Again these results indicate the complex mechanisms involved in cellular chain length. Multiple gene functions may be responsible for the altered length phenotype in *S. sanguinis*.
